# Higher Levels of Neutralizing Antibodies against KSHV in KS Patients Compared to Asymptomatic Individuals from Zambia

**DOI:** 10.1371/journal.pone.0071254

**Published:** 2013-08-14

**Authors:** Pankaj Kumar, Nithal Y. Kuwa, Veenu Minhas, Clemence Marimo, Danielle M. Shea, Chipepo Kankasa, Charles Wood

**Affiliations:** 1 Nebraska Center for Virology and the School of Biological Sciences, University of Nebraska-Lincoln, Lincoln, Nebraska, United States of America; 2 Department of Pathology and Microbiology, University of Zambia School of Medicine, Lusaka, Zambia; 3 Department of Paediatrics, University of Zambia School of Medicine, Lusaka, Zambia; Hannover Medical School, Germany

## Abstract

Kaposi sarcoma-associated herpesvirus (KSHV) is the etiologic agent for Kaposi Sarcoma (KS), the most common cancer diagnosed in HIV- infected patients. The role of neutralizing antibodies in KS pathogenesis and in KSHV infected individuals is not clearly understood. The goal of this study was to investigate and compare the prevalence and titers of neutralizing antibodies in plasma samples from KS patients and KSHV infected asymptomatic individuals from Zambia, a KS endemic region in sub-Saharan Africa. Plasma samples (N = 267) consisting of KS patients (group 1) and asymptomatic individuals (group 2) were collected from Lusaka, Zambia. A flow cytometry based quantitative neutralization assay utilizing recombinant KSHV expressing GFP was used to detect KSHV neutralizing antibodies. Our results show that the overall prevalence of neutralizing antibodies in KS patients (group 1) was 66.7% which was significantly higher than the prevalence of 6.5% present in KSHV infected asymptomatic individuals (group 2). Total antibody titers as well as neutralizing antibodies titers were found to be significantly higher among KS patients. It is likely that higher neutralizing antibodies prevalence and titers in KS patients result from higher levels of antigenic stimulation over time. This study is first to compare prevalence and titers of neutralizing antibodies in participants with and without disease from a KSHV endemic region.

## Introduction

Human herpesvirus-8 (HHV-8) also known as Kaposi’s sarcoma-associated herpesvirus (KSHV) is the etiological agent of Kaposi’s sarcoma (KS) and at least two other malignancies; primary effusion lymphoma and multicentric Castleman’s disease [1], [2], [3]. KS is a multifocal neoplasm characterized by angiogenesis, proliferation of spindle cells, edema and occasional dissemination into visceral organs [4], [5]. KS predominantly occurs in immunosuppressed individuals and is one of the most common malignancies associated with HIV infection. KSHV infection and KS prevalence is low in general population in the US but is high in endemic regions such as the ‘KS belt’ in sub-Saharan Africa. Zambia is a part of the “KS belt” where KS is endemic and a dramatic increase in the incidence of KS in adults and children has also been reported with the advent of the HIV epidemic [6], [7], [8], [9]. The fact that KSHV causes tumors in immunocompromised patients underscores the importance of a functional immune system in controlling KSHV infection. However, little is known about the role of immune response in the development of KS, especially in sub-Saharan Africa which is currently experiencing an HIV epidemic.

Neutralizing antibodies are an important component of the humoral immune response and have been implicated in controlling the progression of herpesvirus-associated disease [10]. Their role in controlling KSHV infection and KSHV-associated disease is still not clear. Till now there have been only two studies that have investigated the prevalence and titers of neutralizing antibodies in KS patients or in KSHV infected asymptomatic individuals. Both reports have focused on a small number of KS patients from the US. One has reported that KS patients had lower titers of neutralizing antibodies compared to asymptomatic individuals irrespective of their HIV status [11], while the other study found no significant difference between the two groups [12]. In addition, the overall prevalence of neutralizing antibodies in KS patients or asymptomatic subjects was found to be low and comparable between the two groups [12]. A lack of comprehensive studies makes it further more difficult to interpret the role of neutralizing antibodies in KSHV infection, especially in populations where KS is endemic.

We and other groups have earlier reported that in sub-Saharan Africa, the seroprevalence of KSHV among adults is between 29% to 48% which is relatively higher compared to the prevalence in Western countries [13], [14]. To date, there has not been any study to investigate the prevalence of neutralizing antibodies in KS patients or in KSHV infected asymptomatic subjects in an endemic area. Whether the neutralizing antibody profile in endemic areas is similar to previous studies conducted in the US is not known. Therefore, the aim of this study was to compare the prevalence and titer of neutralizing antibodies against KSHV in KS patients and in asymptomatic individuals in an endemic region, Zambia.

## Materials and Methods

### Study Subjects

A total of 267 plasma samples collected from patients at University Teaching Hospital, Lusaka, Zambia, were used in this study. These plasma samples were divided into two groups based on the presence or absence of clinical KS in the patients. Group 1 included plasma samples collected during 2011 from 36 KS patients who were seropositive for KSHV. All patients were clinically diagnosed with KS and the initial diagnosis was confirmed by a biopsy report. Group 2 comprised of plasma samples from 231 asymptomatic individuals with KSHV positive serostatus but without clinical KS. These samples are a part of a larger ongoing cohort study conducted from 2004 to 2009 to investigate KSHV transmission within families and were collected from caregivers who brought their child to the study clinic as described previously [15]. Written informed consent was obtained from all study participants. Additionally, KSHV seronegative plasma samples from anonymous healthy blood donors from local blood bank in Lincoln, NE were used for assay controls. The study was approved by the Institutional Review Board of the University of Nebraska-Lincoln and the ethics board of the University of Zambia.

### KSHV and HIV Serology

The KSHV serostatus of participants was determined by monoclonal-enhanced immunofluorescence assay (mIFA) as described previously [16]. All positive plasma samples were diluted further and tested by mIFA to determine the total KSHV antibody titer of each sample. mIFA involved the use of tetradecanoyl phorbol acetate (TPA) stimulated BC-3 cells that were fixed and permeabilized on to teflon coated slides. To reduce subjectivity in observing specific fluorescence, slides were read independently by two laboratory workers. TPA stimulated BC3 cell slides were used to determine total KSHV titer because BC3 cells are latently infected with KSHV and upon stimulation express all the KSHV lytic genes.

The HIV-1 status was determined, as recommended by the Zambian Ministry of Health, using HIV-1/HIV-2 Abbott determine (Abbott Laboratories) and confirmed using Unigold kit (Trinity Biotech).

### Cell Culture and rKSHV.219 Production

Human embryonic kidney cell line, HEK293, was grown in Dulbecco modified Eagle medium (DMEM) supplemented with 10% fetal calf serum. Vero cells stably expressing KSHV tagged green fluorescent protein (Vero.219) were maintained in DMEM containing 10% fetal calf serum, and puromycin (6 µg/ml) for selection. HEK293 cells were purchased from ATCC, Manassas, VA. Vero.219 cells were a kind gift of Dr Jeff Vieira (University of Washington, Seattle) [17].

Recombinant KSHV expressing GFP (rKSHV.219) was produced and titered as described previously by stimulating latently infected Vero.219 cells [17]. Briefly, Vero.219 cells were stimulated with baculovirus expressing KSHV RTA at room temperature for 4 hours. Vero.219 cells were further stimulated with 1.25 mM sodium butyrate in medium supplemented with puromycin for 24 hours. The medium was replaced with fresh medium after 24 hrs. Supernatant containing virus was collected 72 hours post stimulation, filtered and stored in the vapor phase of liquid nitrogen. To titer rKSHV.219 virus, 293 cells were grown to 70–80% confluency in 96 well plates. The supernatant containing virus was diluted 7 times at 4 fold dilutions starting at a 1∶12 dilution. The diluted virus was added to 293 cells in triplicate wells and incubated for 48 hours. The number of cells expressing GFP in each well was counted using a fluorescent microscope. The titer was calculated using the Spearman-Karber formula. Viral titers of up to 10^5^ infectious units/ml were used for neutralization assay.

### Neutralization Assay

Neutralization assay was performed based on the method previously described by Kimball et al but with some modifications [11]. Neutralization assay was carried out in triplicate wells of 96-well culture plates. Briefly, heat inactivated plasma (56°C for 30 min) was incubated at a 1∶50 dilution with rKSHV.219 virus for 1 hr at 37°C. Pre-plated 293 cells were infected with virus-plasma mixture, centrifuged (400×g, 20 minutes) and subsequently incubated at 37°C for 72 hours. The level of infection was monitored by the number of cells expressing GFP using an inverted fluorescent microscope and quantitated using a flow cytometer (BD Biosciences) 72 hours post infection. A plasma sample was considered positive for the presence of neutralizing antibodies when 50% or higher inhibition in infectivity was observed as compared to negative control plasma.

**Figure 1:**
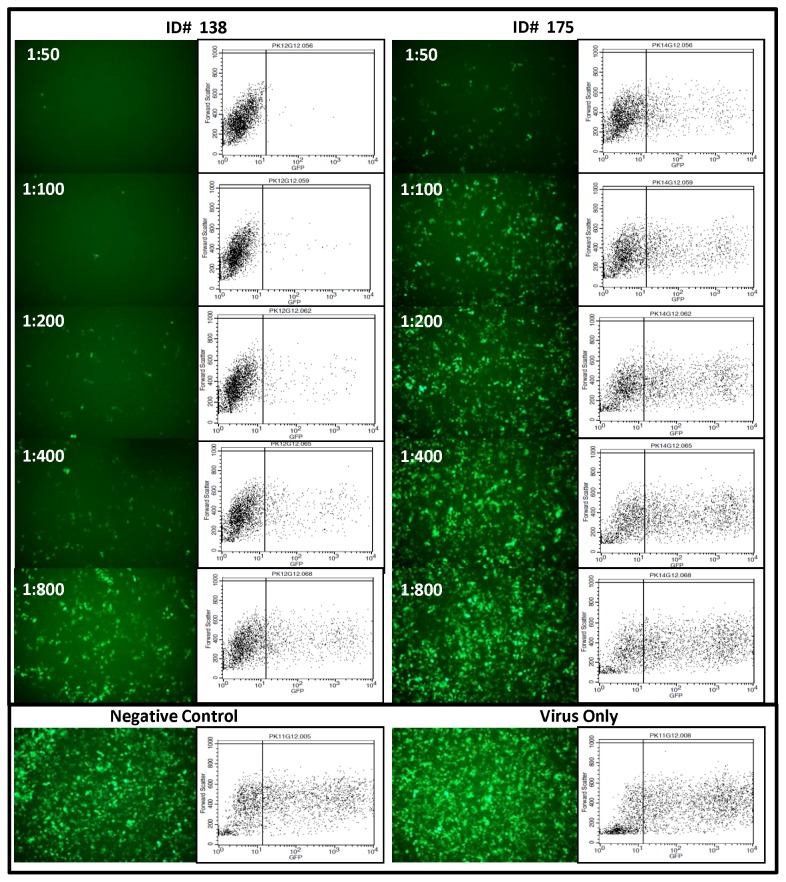
KSHV Neutralization Assay. Fluorescent pictographs of 293 cells infected with rKSHV.219 incubated with serially diluted plasma samples (1∶50, 1∶100, 1∶200 and 1∶800) from two Kaposi’s sarcoma patients (ID 138 and 175) who were neutralizing antibody positive. ID 138 had comparatively higher titer neutralizing antibodies as compared to ID 175. Parallel flow cytometry scatter plots are also presented. Negative control (KSHV and KS negative plasma at a dilution of 1∶50) and virus only control are shown at extreme right.

Plasma samples that were positive for neutralizing antibodies were further titrated by 2-fold dilution starting at 1∶50 to 1∶800. IC_50_ (50% of inhibitory concentration) was calculated using GraphPad Prism version 5.05 (La Jolla, CA). Fluorescence images from neutralization assay of two representative samples from group I (ID#175 had lower and ID#138 had higher neutralizing antibody titer) along with their parallel scatter plots are shown in Figure 1. With increasing serial dilution of plasma, viral infectivity (indicated by cells expressing GFP) increased and gradually approached to that of the input virus or virus incubated with KSHV negative plasma. The level of viral infectivity in the presence of KSHV negative plasma was similar to that of input virus (without plasma). No significant difference was observed in the number of cells expressing GFP, with increasing dilution of KSHV negative plasma (data not shown).

**Figure 2:**
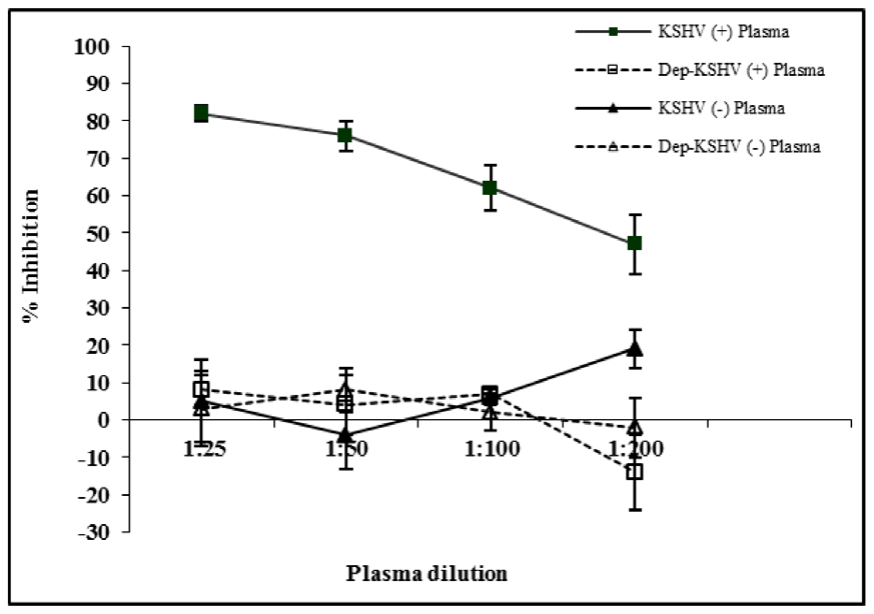
KSHV inhibition is antibody mediated. IgG antibodies from KSHV positive and negative plasma were depleted using protein G sepharose beads. Plasma was serially diluted and incubated with rKSHV.219 before infecting 293 cells. Percent inhibition at each plasma dilution was calculated by normalizing sample infectivity to infection with virus in the absence of plasma.

To ensure that the inhibition is antibody mediated and is not due to a non-specific inhibitory factor that may have inhibited KSHV infection. IgG antibodies were specifically depleted from the plasma by incubating it with protein G sepharose beads overnight (Figure 2). The ability of IgG depleted plasma to neutralize rKSHV.219 virus was carried out by neutralization assay as described above. No significant difference was observed in the level of viral inhibition between IgG depleted and non-depleted KSHV negative plasma. The level of viral inhibition in the presence of IgG depleted KSHV positive plasma were similar to those of the non-depleted and depleted KSHV negative plasma. With the KSHV positive plasma, viral inhibition was markedly reduced with increasing plasma dilution.

### Evaluation of Data and Statistical Analysis

Neutralization assay was performed in triplicate and a sample was designated positive or negative (at plasma dilution 1∶50) for the presence of neutralizing antibodies if similar results were obtained the first two times the assay was performed. Initially, each sample was tested twice and the result was considered indeterminate if concordant results were not observed. Subsequently, the assay was repeated a third time. Based on the third result the sample was considered positive or negative. None of the samples in group 1 were tested a third time because 100% concordance was achieved in all the samples. In group 2, 9 out a total of 231 samples were tested a third time because concordant results were not observed after duplicate testing.

Average percent infectivity was calculated and a positive neutralizing antibody outcome was defined as ≥50% reduction in infectivity as compared to the KSHV seronegative control plasma. To determine percent inhibition of viral infection, sample data was normalized to input virus infection and calculated as: Normalized Percent Infectivity = (Sample percent infectivity/Virus only percent infectivity)×100. The percent inhibition was calculated as: (100 minus normalized percent infectivity). To calculate neutralizing antibody titers, the inverse values of the last positive dilution were transformed into log_2_ values and geometric mean titers (GMT) were calculated for each group. P-values were calculated using Mann-Whitney test.

The difference in prevalence of neutralizing antibodies was measured by chi-square test or Fisher’s exact test. Correlation coefficient between neutralizing antibody titers and total KSHV antibody titers were calculated using SAS (v9.2) (PROC CORR). All tests were 2-tailed and p-values <0.05 were considered significant. Power and sample size estimation was done using Sample Power 2 (SPSS). All other statistical analyses were performed using GraphPad Prism (v5.05) and SPSS (v19).

## Results

### Characteristics of Study Cohort

Table 1 summarizes the characteristics of the study population (N = 267) that was divided into two groups. Briefly, all group 1 patients (n = 36) had clinical KS and were also seropositive for HIV. The median CD4 T cell count and HIV viral load was 157 cells/mm^3^ and 143 copies/ml of plasma, respectively. Group 2 (n = 231) comprised of KSHV seropositive asymptomatic individuals. The HIV-1 seroprevalence among this group was 46.7% and the median CD4 T cell count of HIV positive individuals (n = 108) was 319 cells/mm^3^. HIV viral load was not available for this group of subjects due to unavailability of viral load testing in Zambia at the time of study sample collection. The median age for both these groups was comparable with median age of 33 years for group 1 and 31 years for group 2 samples.

**Table 1:** Characteristics of the study population.

	Group 1	Group 2
Characteristic	(KS Samples)n (%)	(Asymptomatic Individuals)n (%)
**Total Number**	36	231
**Gender**		
Male	23 (63.9)	104 (45.0)
Female	13 (36.1)	127 (55.0)
**Median Age** **(years)**	33	31
**HIV Status**		
Positive	36 (100)	108 (46.7)
Negative	0	123 (53.3)
**Median CD4 Count (Range)**	157 (0–1116)	319 (42–1100)
**Median HIV viral load (Range)**	143 (50–100,000)	NA

NA – Data not available.

### Prevalence of Neutralizing Antibodies

**Figure 3:**
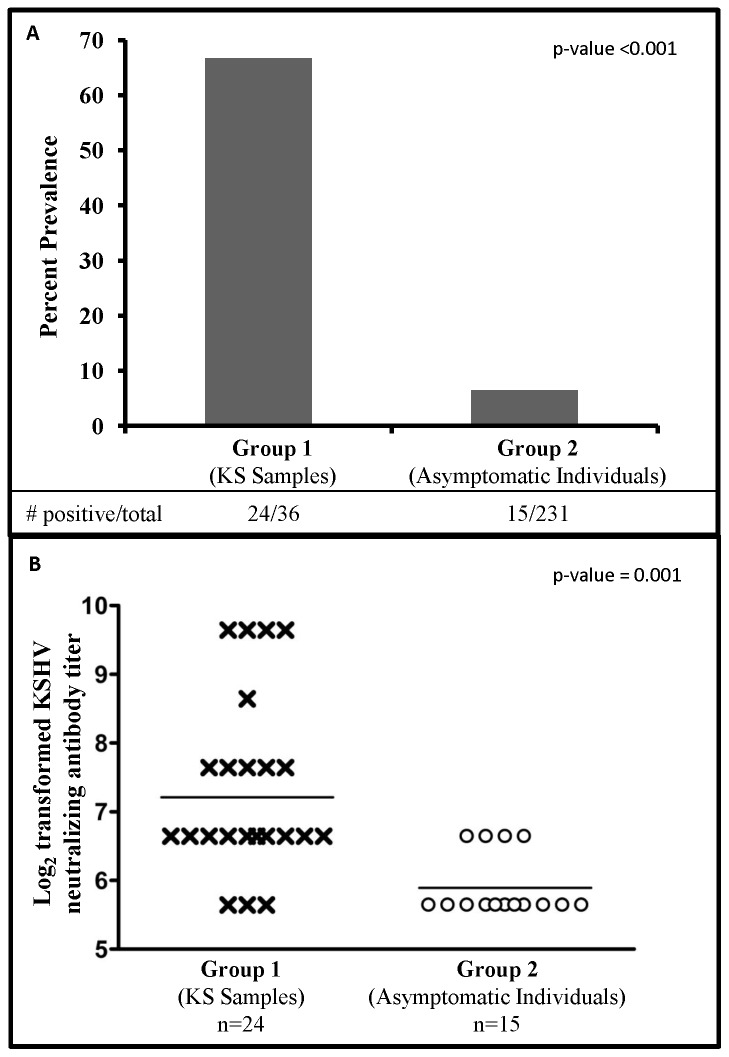
Prevalence and titer of KSHV neutralizing antibodies in group 1 and 2 samples. **A:** Percent prevalence of neutralizing antibodies in group 1 (KS samples) and group 2 (asymptomatic individuals). **B:** KSHV neutralizing antibody titers (log_2_ transformed) in samples from group 1 (KS samples) and group 2 (asymptomatic individuals) participants. GMT of neutralizing antibodies in each group is also marked.

As shown in Table 2 and Figure 3A, the overall prevalence of neutralizing antibodies in group 1 (KS samples) was 66.7% (24/36) and in group 2 (asymptomatic individuals) was 6.5% (15/231) (p-value <0.001). Table 2 compares the study participant characteristics of samples that were positive for neutralizing antibodies in group 1 vs. group 2. We did not observe an association between gender and the presence of neutralizing antibodies in group 1 vs. 2 samples (p-value = 0.18). However, we observed a significant association between age and the prevalence of neutralizing antibodies in group 1 vs. group 2 samples (p-value = 0.004). In group 1, a majority of neutralizing antibody positive patients were older (>30 years) but in group 2, there was no association between age and the prevalence of neutralizing antibodies. We observed neutralizing antibodies in 4/108 (3.7%) HIV positive asymptomatic individuals as compared to 11/123 (8.9%) HIV negative asymptomatic individuals. Overall, the neutralizing antibody prevalence was found to be significantly higher in KS patients as compared to asymptomatic individuals but we could not directly compare group 1 and 2 samples based on the HIV status because all samples in group 1 were HIV positive. We also could not compare the neutralizing antibody positive samples between group 1 and 2 based on CD4 counts because of incomplete data for group 2 samples.

**Table 2:** Study population characteristics: Comparison of samples who elicited a neutralizing antibody response in group 1 and group 2.

	Neutralizing Antibodies Present in		
Characteristic	Group 1(KS Samples)Number positive/total (%)	Group 2(Asymptomatic Individuals)Number positive/total (%)	p-value**
**Total**	24	15	
**Gender**			0.18
Male	17/23 (73.9)	7/104 (6.7)	
Female	7/13 (53.8)	8/127 (6.3)	
**Age** *			0.004
≤30 years	3/12 (25.0)	9/113 (7.9)	
>30 years	20/23 (87.0)	6/118 (5.1)	
**HIV Status**			ND
Positive	24/36 (66.7)	4/108 (3.7)	
Negative	0	11/123 (8.9)	
**CD4 Count** *			ND
≤200 cells/mm^3^	14/22 (6.4)	4/20 (20.0)	
>200 cells/mm^3^	8/12 (66.7)	0/64	

* Some participant characteristics had missing values.

** p value from chi-square test of overall association between sample type (group 1 vs. group 2) and study participant characteristics.

ND – Not determined.

Since there was a significant difference in the prevalence of neutralizing antibodies in group 1 and group 2 samples, all neutralizing antibody positive samples were further titrated to investigate whether there were differences in the neutralizing antibody titers within these groups. Figure 3B compares the GMT of neutralizing antibodies of all samples in group 1 and 2 that had detectable neutralizing antibodies. This data clearly demonstrates that the GMT of neutralizing antibodies in group 1 (KS samples) was significantly higher as compared to group 2 (asymptomatic individuals) (p = 0.001). We also compared the neutralizing antibody titers in males vs. females in each group and found no significant difference between them (data not shown).

**Figure 4:**
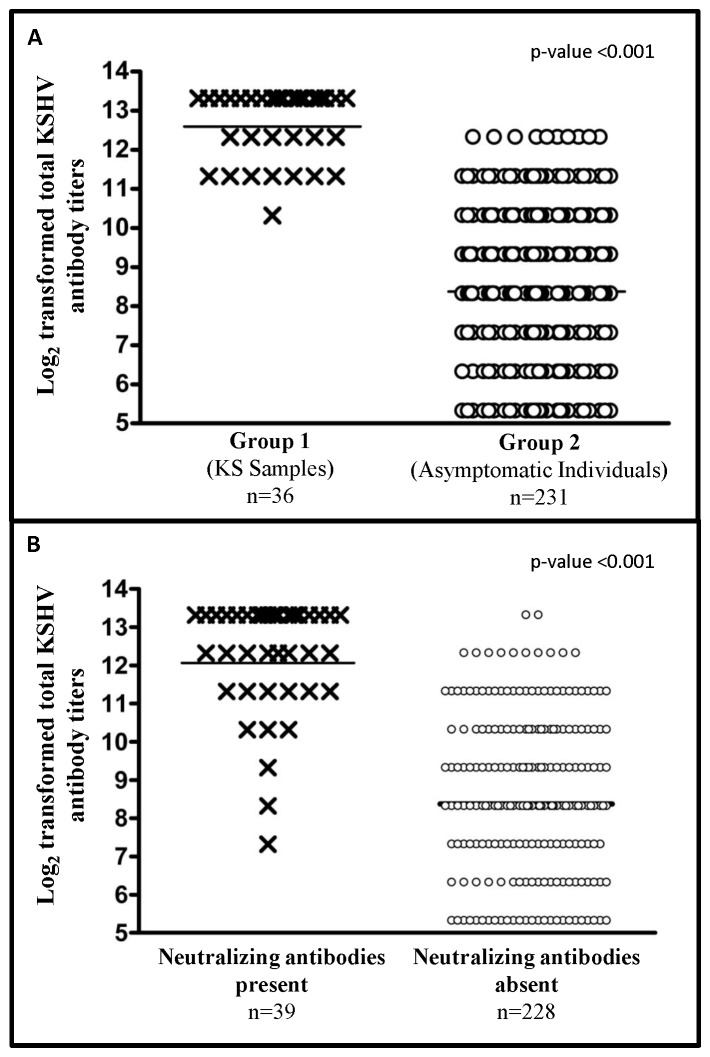
Total KSHV antibody titers in samples. Comparison of total KSHV antibody titer (log_2_ transformed) in; **A:** samples from group 1 (KS samples) and group 2 (asymptomatic individuals) participants and; **B:** all samples that have neutralizing antibody present or absent. GMT of KSHV antibody titers in each group is also marked.

Total KSHV antibody titers were also determined for the entire panel of 267 samples to investigate whether neutralizing antibody titers were associated with total KSHV antibody titers. Figure 4A compares the total KSHV antibody titers of all the subjects in group 1 and group 2. It is clear that GMT of KSHV antibodies in group 1 samples were significantly higher as compared to group 2 samples (p-value <0.001). KSHV antibody titers in samples positive for neutralizing antibodies (n = 39, 24 in group 1 and 15 in group 2) were also compared to samples that did not have detectable neutralizing antibodies (n = 228). This analysis revealed that GMT of KSHV antibodies in neutralizing antibody positive samples were also significantly higher as compared to samples that had no detectable neutralizing antibodies (p-value <0.001) (Figure 4B). Correlation between neutralizing antibody titers and total KSHV antibody titers was also determined and revealed a positive correlation between the two titers (correlation coefficient = 0.47). This positive correlation was also present when samples from each group were tested alone (correlation coefficient for group 1 = 0.33 and for group 2 = 0.40).

## Discussion

This is the first study to compare the prevalence and titers of neutralizing antibodies in plasma samples from KS patients and asymptomatic individuals from an endemic area. In the current study we have used a modified version of a previously reported infectivity assay to detect neutralizing antibodies against KSHV. This assay utilizes a GFP-tagged recombinant KSHV and employs flow cytometry to quantitate the infected cells as a readout system. The quantitative strength of our flow cytometry-based neutralization assay gave us an accurate and high throughput measure of the neutralizing antibody titers of infected individuals. To achieve high specificity, we defined plasma neutralization as 50% or higher inhibition in infectivity compared to control plasma which was KSHV negative.

The findings of this study clearly show that the prevalence and titers of neutralizing antibodies in group 1 (KS patients) were significantly higher as compared to group 2 (asymptomatic individuals) (Figure 3A). Our findings are different from the study reported by Kimball et al who demonstrated that patients in the US diagnosed with AIDS-KS display lower titers of neutralizing antibodies despite having higher levels of anti-KSHV antibodies [11]. This difference could be attributed to different study populations that have vastly different KSHV prevalence in the general population. Our previous research in Zambia with the same study population has shown that children get infected with KSHV at a very early age, in contrast to the situation in US where the seroprevalence of KSHV during childhood is very low. It is likely that neutralizing antibody titer increases over the course of KSHV infection and this increase is driven by higher levels of antigenic stimulation i.e. viral load as has been previously reported in KS patients [18], [19], [20]. We hypothesize that infection at an early age followed by intermittent reactivation of latent virus over time may result in extended exposure to viral antigens in an endemic population. Also, it would provide time for the expansion of B-cells producing neutralizing antibodies as the maturation of humoral response against KSHV occurs in the infected individual. Recent studies investigating the determinants of neutralizing antibody response in HIV infection support the idea that production of neutralizing antibodies requires continuous antigenic stimulation and usually take years [21], [22].

Our finding that KS patients have high prevalence and increased titers of neutralizing antibodies should not be interpreted to mean that neutralizing antibodies are not protective in case of KSHV infection. It is quite possible that neutralizing antibodies have very different roles during primary KSHV infection and later on during persistent long term infection. In chronically infected patients neutralizing antibodies may have a limited role only in controlling the spread of virus. However during primary infection, neutralizing antibodies can prevent KSHV infection if they are present at the time of exposure as would be the case if elicited by a vaccine prior to primary infection. A prophylactic vaccine that can strongly induce neutralizing antibodies could be useful to prevent infection in KSHV endemic populations.

It is well established that HIV-infected individuals are at a significantly higher risk for developing KS as opposed to HIV-1 negative individuals [23]. KSHV seroprevalence is highest among individuals who are HIV infected compared to uninfected individuals [24]. A recent study has shown that active replication of KSHV was significantly associated with a low number of CD4+cells in HIV positive antiretroviral therapy-naïve males, thus confirming the importance of immune response in controlling KSHV replication [25]. Thus far, no correlation has been reported between the prevalence of KSHV neutralizing antibodies and HIV status of asymptomatic individuals. Unfortunately, the number of neutralizing antibody positive individuals from HIV+ and HIV- asymptomatic individuals (group 2) were too small to establish a meaningful correlation. The association between neutralizing antibodies and HIV status needs to be studied in a larger cohort of asymptomatic individuals.

It has been reported previously that the titer of KSHV antibodies is higher in KS patients as compared to patients who are KSHV infected asymptomatic patients [26], [27]. We observed similar results in the present study that the GMT of KSHV antibodies were significantly higher in group 1 as compared to group 2 (Figure 4A). Moreover, we observed that the KSHV antibody titers in samples which had detectable neutralizing antibodies were significantly higher as compared to samples that did not have neutralizing antibodies (Figure 4B). We also observed a positive correlation between KSHV antibody titers and neutralizing antibody titers. This supports our hypothesis that there is a slow maturation of the immune response against KSHV, over a period of time to result in a concurrent increase in both neutralizing antibody titers and KSHV antibody titers.

A limitation of this study was a lack of KSHV viral load information due to limited availability of plasma samples. Also the cross-sectional design of our study makes it difficult to predict the duration and levels of neutralizing antibody response over a period of time. Although hard to execute, a longitudinal follow up of infected individuals would provide clues about the existence of a temporal relationship between viral load and neutralizing antibody response. It would be equally important to investigate the kinetics of neutralizing antibody response from the time of seroconversion to KS diagnosis and determine whether neutralizing antibodies against KSHV can serve as prognostic markers for disease progression.

In summary, by using a standardized quantitative neutralization assay we have the investigated the prevalence and titers of neutralizing antibodies in KS patients and asymptomatic individuals from an endemic region. To our knowledge, this is first study of its kind from a KS endemic region. Our results show that there is low prevalence of neutralizing antibodies in asymptomatic individuals, in contrast to high prevalence and higher titers of neutralizing antibodies in KS patients. In future, we plan to investigate the development of neutralizing antibodies on a larger longitudinal cohort to better define the role of neutralizing antibodies in the pathogenesis of KSHV and the development of KS.
